# Four decades of new vascular plant records for Greenland

**DOI:** 10.3897/phytokeys.145.39704

**Published:** 2020-04-10

**Authors:** Christian Bay

**Affiliations:** 1 Institute for Bioscience, Aarhus University, Frederiksborgvej 399, 4000 Roskilde, Denmark Aarhus University Roskilde Denmark

**Keywords:** Vascular plants, flora, Greenland, phytogeography, distribution limits

## Abstract

Records of new species of vascular plants in Greenland from the last four decades are presented and new phytogeographical data leading to extension of the known distribution limits in Greenland are discussed. Since the publication of the latest edition of the Flora of Greenland in 1978 (Böcher et al. 1978) fieldwork by Greenland Botanical Survey and other expeditions have taken place especially in West and East Greenland and in many remote areas in North and Northeast Greenland. This paper serves as an update of the Flora of Greenland. Twenty species, one subspecies and one new forma have been added to the flora of Greenland: *Carex membranacea* Hook., *Carex miliaris* Michx., *Carex rhomalea* (Fernald) Mack., *Equisetum hyemale* L., *Festuca edlundiae* S. Aiken, Consaul and Lefkovich, *Festuca groenlandica* (Schol.) Frederiksen, *Festuca saximontana* Rydb., *Galium verum* L., *Geum rossii* (R. Br.) Ser., *Papaver cornwallisense* D. Löve, *Papaver dahlianum* Nordh., *Papaver labradoricum* (Fedde) Solstad and Elven, *Papaver lapponicum* (Tolm.) Nordh., Pedicularis sudetica Willd. ssp. albolabiata Hult., *Poa flexuosa* Sm., *Puccinellia bruggemanni* Th. Sør., *Ranunculus subrigidus* W.B. Drew., *Silene vulgaris* (Moench) Garcke, *Trientalis europaea* L. and *Veronica officinalis* L. in addition to one subspecies Phippsia algida (Sol.) R. Br. ssp. algidiformis (H. Sm.) Löve and Löve. The viviparous form of Poa hartzii f. prolifera has been reported for the first time in Greenland. Presently, the total number of vascular plant species in Greenland is 532. 89 new northern and 28 new southern distribution limits are presented and 26 species are new to the flora province East Greenland, whereas 15 species are new to West Greenland. The numbers of new species to flora provinces North and South Greenland are 14 and one, respectively.

## Introduction

Greenland is the largest island in the world, extending from c. 60° to c. 83° northern latitude, and it includes all the Arctic bioclimatic zones (Raynolds et al. 2019) from the subarctic zone in continental areas in southernmost Greenland to the polar desert zone in coastal areas of North Greenland (Bay 1997). Ice-free areas have a varying width of up to 200 km from the outer coast to the Inland Ice. This large variation in climate from coastal to inland areas, in addition to large differences in regional geology and soils, gives rise to a large number of biological niches. Despite this fact only 532 species of vascular plants are known from Greenland, which is a low number considering the size and distribution of the island in all the bioclimatic zones of the Arctic. The immigration of species is restricted because of the remoteness of Greenland to neighboring territories in North America and Eurasia. The species number in the neighboring arctic territories in Canada, Russia, and Norway, which covers larger or smaller areas compared to Greenland, is 375 (Gillespie et al. 2015), 1691 (Sekretareva 2004) and 184 (Alsos et al. 2017), respectively. Generally, the Arctic flora is young with low species diversity, low endemicity, and is little influenced by alien species (Daniëls et al. 2013). Recently an updated red list of Greenland has been published including all endemic species of vascular plants (Boertmann and Bay 2019).

An updated flora is an important baseline information when assessing the changes in number and species composition in a changing climate in the near future.

The vascular plant flora of Greenland has been studied intensively during the latest decades and three phytogeographical papers have been published based on material in the Copenhagen herbarium (C) and other herbaria. South Greenland was studied by Feilberg (1984), North Greenland by Bay (1992), and West Greenland by Fredskild (1996); in addition the material from East Greenland is under preparation by C. Bay. Taxonomical revisions of species complexes by Solstad and Elven (*Papaver* complex) and Myhre Pedersen and Elven (*Carex saxatilis* complex) have added a few species.

The botanical exploration of Greenland started in the easily accessed areas in South and West Greenland, whereas the exploration of the remote areas in North and Northeast Greenland followed decades later. Greenland Botanical Survey at University of

Copenhagen carried out floristic and vegetation studies in most parts of Greenland during the period 1962–1998 (Bay et al. 2017).

Since the publication of the Flora of Greenland (Böcher et al. 1978), one update was made 25 years ago (Bay 1993) that summarized the total number of vascular plant species to 513. During the latest 15 years the total number of vascular plants has been increased by nineteen species giving a total of 532 species. The present paper concerns all the finds of new taxa to Greenland and the new distributional records since the Flora of Greenland was published forty years ago.

An updated flora is an important baseline information when assessing the changes in number and species composition in a changing climate in the near future. An update of the none-native vascular plants in the Arctic has been published recently (Wasowicz et al. 2020).

## Methods and materials

Fieldwork in recent years by the Greenland Botanical Survey (GBS) and others (Table 1) have resulted in finds of species new to the flora of Greenland and have extended the knowledge on northern and southern distribution limits for many species. Especially, fieldwork in North and Northeast Greenland in the eighties and nineties, which have been inaccessible for decades because of their remoteness, has added new phytogeographical knowledge. In addition valuable data have been collected from mid-West Greenland when Jon Feilberg and Vilhelm Dalgaard were leaders of the Arctic station on the island Disko.

Several botanist have contributed with important knowledge to the flora of Greenland. B. Fredskild worked in Northeast Greenland ten summers in the period 1982–1996 and more than twenty seasons in West Greenland, while C. Bay worked 29 summers in Greenland, mostly in high arctic areas.

Other important contributors are P. Gelting, G. Seidenfaden, T. Sørensen, F. Rune, G. Halliday, R. Corner, R. and S. David, H. Lang and F. Schwartzenbach, who have contributed with important collections from West, East and North Greenland to the Greenland herbarium of University of Copenhagen. A large number of specimens was collected, identified and stored in C. This information is gathered in the yearly Greenland Botanical Survey reports and is the main basis for this paper together with specimens from other herbaria and recent finds. The nomenclature is according to Böcher et al. 1978.

## Results

Twenty species, one subspecies and a new forma have been added to the flora of Greenland since the last edition of The Flora of Greenland (Böcher et al. 1978): *Carex membranacea* Hook., *Carex miliaris* Michx., *Carex rhomalea* (Fernald) Mack., *Equisetum hyemale* L., *Festuca edlundiae* Aiken, Consaul and Lefkovich, *Festuca groenlandica* (Schol.) Frederiksen, *Festuca saximontana* Rydb., *Galium verum* L., *Geum rossii* (R. Br.) Ser., *Papaver cornwallisense* D. Löve, *Papaver dahlianum* Nordh., *Papaver labradoricum* (Fedde) Solstad and Elven, *Papaver lapponicum* (Tolm.) Nordh., Pedicularis sudetica Willd. ssp. albolabiata Hult., *Poa flexuosa* Sm., *Puccinellia bruggemanni* Th. Sør., *Ranunculus subrigidus* W.B. Drew., *Silene vulgaris* (Moench) Garcke, *Trientalis europaea* L. and *Veronica officinalis* L. In addition one subspecies Phippsia algida (Sol.) R. Br. ssp. algidiformis (H. Sm.) Löve and Löve is a new taxon to the flora of Greenland. The viviparous form of Poa hartzii f. prolifera has been reported for the first time in Greenland. The total number of vascular plant species for Greenland is 532. The results are presented in Table 1.

**Figure 1.**
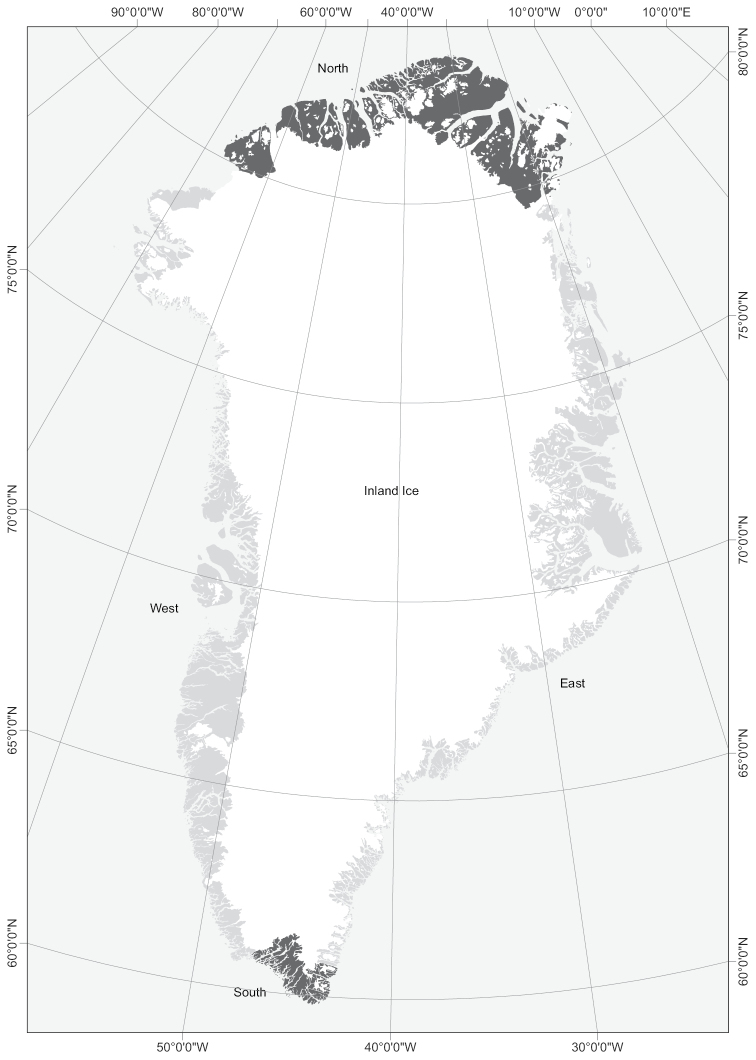
The floristic provinces of Greenland (Böcher et al. 1978).

Of the 532 species in Greenland 89 species (17%) are recorded north of their known northern distribution limit, and 28 species (5%) are recorded south of their known southern limit. Twelve species are new to East Greenland and three are new to West Greenland. The new phytogeographical records are summarized in Table 1 and the floristic provinces are shown in Figure 1.

## Discussion

Generally, the findings of species new to Greenland and extensions of the distribution areas are mostly due to the fact that botanical explorations have taken place in remote areas supported by helicopter and aircrafts, which hitherto had only been accessible by boat. Many inland areas both in West and East Greenland have been investigated in recent decades. The extension of the distribution limits is not considered a result of climate change but rather a result of the intensification of the botanical exploration of remote areas. The fruits or spores of new species have either been brought to Greenland by migrating geese (i.e. *Geum rossii*) or introduced by man (i.e. *Galium verum*, *Silene vulgaris*, *Veronica officinalis*). Table 1 summarize the new phytogeographical data and the most notable finds are annotated.

**Table 1.** New phytogeographical records of vascular plant by the Greenland Botanical Survey and others during 1979–2019. For each species the floristic province, locality, phytogeographical record and the publication or the collector are provided.

Taxon	Floristic province and locality	Phytogeographical records	Reference; Publication/ Collector
*Alchemilla glomerulans* Bus.	East Greenland: Bjørneøer	New north limit: 71°10'N	I. Smart leg. 1980
*Antennaria angustata* Greene	West Greenland: Isua	New south limit: 65°12'N	Bay and Simonsen 2013
*Antennaria canescens* (Lge.) Malte	West Greenland: Melville Bugt. East Greenland: Grandjean Fjord	New north limit in West: 75°25'N; New north limit in East Greenland 75°00'N	Bay 1992 Halliday 2019a
*Antennaria porsildii* E. Ekmann	East Greenland: Kuhn Ø	New north limit 74°44'N	G. Halliday leg. 1990
*Arabis arenicola* (Richards) Gel.	East Greenland: Lindemann Fjord	New north limit: 74°38'N	G. Halliday leg. 1980
*Arctostaphylos alpina* (L.) Spreng.	East Greenland: Kuhn Ø	New north limit: 75°N	R. Corner and G. Halliday leg. 1990
*Arnica angustifolia* M. Vahl	North Greenland: Frigg Fjord	New north limit: 83°04'N	Bay 1992
*Arenaria humifusa* Wahlenb.	East Greenland	New north limit: 75°52'N New south limit: 70°20'N	Halliday 2019a
*Arctagrostis latifolia* (R.Br.) Griseb.	East Greenland: Hjørnedal	New south limit: 70°19'N	Halliday 2019a
*Bartsia alpina* L.	East Greenland: Kap Beaupré	New north limit: 68°54'N	Gilg et al. 2005
*Betula nana* L.	East Greenland: Adolf Jensen Land	New north limit: 76°16'N	Halliday 2019a
*Botrychium lunaria* (L.) Swe.	East Greenland: Gaus Halvø	New north limit: 73°18'N	O. Gilg leg. 2005
*Braya thorild-wulffii* Ostf.	West Greenland: Disko	New south limit: 69°54'N	Fredskild 1996
Calamagrostis lapponica (Wbg.) Hartm. var. groenlandica Lge.	West Greenland	New south limit: 66°13'N	Bay and Simonsen 2009
*Calamagrostis neglecta* (Ehrh.) Gaertm., Mey. & Scherb.	East Greenland: Kuhn Ø	New north limit: 74°44'N	R. Corner and G. Halliday leg. 1990
*Calamagrostis purpurascens* R. Br.	East Greenland: Skjoldungen district	New south limit: 63°21'N	C. Bay leg. 1992
*Campanula gieseckiana* Vest in R. and S.	East Greenland: Dove Bugt	New north limit: 76°16'N	D. Shaw leg. 2006
*Cardamine pratensis* L.	North Greenland: Nansen Land	New north limit: 82°58'N	Bay 1992
*Carex atrofusca* Schkuhr.	North Greenland: Nansen Land	New to North Greenland: 82°58'N	Bay 1992
*Carex chordorrhiza* Ehrh.	East Greenland: Jameson Land and Tyrolerfjord	New to East Greenland: 71°10'N and 74°30'N	A. Elvebakk leg. 1993, Bay 2015
*Carex glacialis* Mack.	North Greenland: Wulff Land	New north limit: 82°10'N	Bay 1992
*Carex glareosa* Wahlenberg	East Greenland: Kuhn Ø	New north limit: 75°01'N	G. Halliday leg. 1990
*Carex holostoma* Drej.	West Greenland	New south limit: 65°32'N	C. Bay leg. 2009
*Carex lachenalii* Schkuhr.	West Greenland: Etah. East Greenland: Langesø	New north limit in West Greenland: 78°20'N and in East Greenland: 75°49'N	Bay 1992, Halliday 2019a
*Carex macloviana* D´Urv	Northeast Greenland: Adolf Jensen Land	New north limit: 74°38'N	R. Corner leg. 2002
Carex marina Dew. ssp. pseudolagopina (Th. Sør.) Böch.	North Greenland: Nansen Land	New to North Greenland 82°58'N New north limit: 83°16'N	Bay 1991, F. Schwartzenbach leg. 1996
*Carex membranacea* Hook.	West Greenland: Inglefield Land and Fortune Bay	New to Greenland	R. Elven pers. com.
*Carex microglochin* Wbg.	West Greenland: Uvkusigssat Fj., East Greenland: Grandjean Fjord	New north limit in West Greenland: 72°15'N and in East Greenland: 74°57'N	Fredskild 1996, J. Hoodson and C. Wells leg. 1990
*Carex misandra* R. Br.	West Greenland: Isua	New south limit: 65°12'N	Bay and Simonsen 2013
*Carex miliaris* Michx.	West Greenland	New to Greenland	Myhre Pedersen pers. com.
*Carex norvegica* Retz.	East Greenland: Femdalen	New north limit: 75°18'N	Bay 1992
*Carex parallela* (Læst.) Sommerf.	East Greenland: Adolf S. Jensen Land	New north limit: 76°16'N	R. Corner leg. 2006
*Carex rhomalea* (Fernald) Mack.	West and East Greenland	New to Greenland	Myhre Pedersen pers. com.
*Carex rupestris*	North Greenland: Nansen Land	New north limit: 82°58'N	Bay 1992
*Carex rostrata* Stokes	East Greenland: Skjoldungen	New to East Greenland: 63°21'N	C. Bay 1992 leg.
*Carex scirpoidea* Michx.	East Greenland: Kuhn Ø	New north limit: 74°46'N	R. Corner and Halliday leg. 1990.
Carex supina Wbg. ssp. spaniocarpa (Steud.) Hult.	West Greenland: Siorapaluk	New north limit: 77°48'N	Bay 1992
*Cerastium cerastoides* (L.) Britton	East Greenland: Lindemann Fjord	New north limit: 74°38'N	Bay 1992
*Comarum palustre* L.	West Greenland:	New north limit: 68°40'N	Fredskild 1996
*Deschampsia pumila* Ostenf.	East Greenland: Norske Øer	New north limit: 79°03'N	Bay 1992
*Deschampsia alpina* (L.) R. and S.	West Greenland	New north limit: 68°37'N	Fredskild 1996
Diapensia lapponica L. ssp. lapponica	West Greenland: Qaanaaq	New north limit: 77°28'N	Bay 1992
*Diphasiastrum complanatum* (L.) Holub	East Greenland: Milne Land	New to East Greenland 70°35'N	C. Bay leg. 2014
*Draba adamsii* Led.	West Greenland Nugssuaq	New south limit: 70°17'N	Fredskild 1996
*Draba arctica* J. Vahl	East Greenland: Sortekappasset	New south limit: 68°31'N	Halliday 2019b
*Draba cana* Rydb.	South Greenland: Kvanefjeld	New south limit: 61°00'N	C. Simonsen 2014
*Draba crassifolia* Graham	East Greenland: Kuhn Ø	New north limit: 74°46'N	Halliday 2019a
*Draba fladnizensis* Wulf.	East Greenland: Skjoldungen	New south limit: 63°28'N	C. Bay leg. 1992
*Draba subcapitata* Simm.	Tasiilaq dist.	New south limit: 66°55'N	Halliday 2019b
Dryas octopetala L. ssp. punctata (Juz.) Hult.	East Greenland: Amdrup Land	New to North Greenland: 80°49'N	Bay and Fredskild 1994
*Dryopteris assimilis* Walker	East Greenland: Fridtjof Nansens Halvø	New to East Greenland: 64°20'N	K. Gormsen leg. 1968
*Dryopteris filix-mas* (L.) Schott	East Greenland: Skjoldungen	New to East Greenland: 63°21'N	C. Bay 1992 leg.
*Dupontia fisheri* R. Br.	East Greenland: Wollaston Forland	New to East Greenland: 74°29´-74°37'N	Fredskild and Bay 1993
*Dupontia psilosantha* Rupr.	East Greenland: Hochstetter Forland	New north limit: 75°28'N	Bay 1992
*Elymus hyperarcticus* (Polunin) Tzvel.	East Greenland: Jameson Land	New south limit: 70°44'N	Fredskild and Bay 1984
Empetrum nigrum L. ssp. hermaphroditum (Hagerup) Böch.	North Greenland: Mylius Erichsen Land	New north limit and new to North Greenland: 80°20'N	F. Daniëls leg. 1995
*Epilobium anagallidifolium* Lam.	West Greenland: Hareøen East Greenland: Allday dal	New north limit in West Greenland: 70°23'N New north limit in East Greenland 71°44'N	Fredskild 1996 S. Holt leg. 1983
*Epilobium arcticum* Sam.	North Greenland: Frigg Fjord	New north limit: 83°15'N	F. Schwartzenbach leg. 1996
*Equisetum hyemale* L.	East Greenland: Tasiilaq	New to Greenland: 65°53'N	Daniëls and Van Herk 1984
*Erigeron humilis* Graham	East Greenland: Godfred Hansen Ø	New north limit: 76°23'N	Bay 1992
Eriophorum angustifolium Honck. ssp. subarcticum (V. Vassil.) Hult.	West Greenland: Thule district	New north limit: 77°28'N	Bay 1992
*Eriophorum callitrix* Cham.	West and North Greenland: Qaanaaq and Nansen Land	New to West Greenland at 77°28'N and new north limit in North Greenland: 82°58'N	C. Bay 1988, leg. 1989
*Eutrema edwardsii* R. Br.	East Greenland: Sødal	New south limit: 70°42'N	R. Corner leg. 1986
*Festuca baffinensis* Polunin	East Greenland: Kangerlussuaq	New south limit: 68°49'N	Halliday 2019b
*Festuca edlundiae* S. Aiken, Consaul and Lefkovich	East Greenland: Hold with Hope	New to Greenland: 73°28'N	Aiken, Consaul and Lefkovich 1995
*Festuca groenlandica* (Schol.) Frederiksen	Low Arctic Greenland	New to Greenland	Frederiksen 1982
*Festuca saximontana* Rydb.	Low Arctic West Greenland	New to Greenland	Fredskild 1996
*Galium verum* L.	East Greenland: Tasiilaq	New to Greenland	Gartmann 1990
*Gentiana detonsa* Rottb.	East Greenland: Tyrolerfjord; Renland	New north limit: 74°30'N New south limit: 70°26'N	R. and S. David leg. 1992; G. Halliday 1971
*Gentiana nivalis* L.	East Greenland: Geographical Society Ø	New north limit: 72°52'N	D. Shaw leg. 2005
*Geum rossii* (R. Br.) Ser.	East Greenland: Lambert Land	New to Greenland: 79°10'N	Bay 1992
*Gymnocarpium dryopteris* (L.) Newman	East Greenland: Liverpool Ld.	New north limit: 71°08'N	B. Fredskild leg. 1985
*Harrimanella hypnoides* (L.) Coville	East Greenland: Kuhn Ø	New north limit: 74°42'N	Halliday 2019a
Isoëtes echinospora Dur. ssp. muricata (Dur.) Löve and Löve	East Greenland: Skjoldungen	New to East Greenland: 63°21'N	C. Bay leg. 1992
Juncus alpinus Vill. ssp. nodulosus (Wbg.) Lindm.	West Greenland	New north limit: 69°35'N	Fredskild 1996
*Juncus arcticus* Willd.	East Greenland: Nørlund Land	New north limit: 75°51'N	G. Halliday leg. 1980
*Juncus castaneus* Sm.	North Greenland: Nansen Land	New north limit: 82°58'N	Bay 1992
*Juncus filiformis* L.	East Greenland: Skjoldungen	New to East Greenland: 63°21'N	C. Bay leg. 1992
*Juncus ranarius* Perr. and Song.	East Greenland: Liverpool Ld.	New north limit: 71°08'N	B. Fredskild leg. 1985
*Juncus trifidus* L.	West and East Greenland	New north limits in West: 72°40'N and in East: 74°44'N	Fredskild 1996 Bay 1992
*Kobresia simpliciuscula* (Wbg.) Mack.	North Greenland: Warming Land	New to western North Greenland	Bay 1992
*X Ledodendron vanhoeffeni* (Abromeit) Dalgaard and Fredskild	West Greenland: Paradisdalen	New south limit: 66°30'N	Fredskild 1996
*Ledum groenlandicum* Oed.	West Greenland: Egedesminde	New north limit: 68°42'N	Fredskild 1996
Ledum palustre L. ssp. decumbens (Ait.) Hult.	West Greenland: Mac Cormick Fjord	New north limit: 77°44'N	Bay 1992
*Loiseleuria procumbens* (L.) Desv.	East Greenland: Jameson Land	New north limit: 70°44'N	C. Bay leg. 1983
*Luzula arctica* Blytt	West Greenland: Isua	New south limit: 65°12'N	Bay and Simonsen 2013
*Luzula multiflora* (*Retz.*) Lej.	East Greenland: Geographical Society Ø	New north limit: 72°42'N	D. Shaw leg. 2005
*Luzula parviflora* (Ehrh.) Desv.	East Greenland: Skjoldungen	New to East Greenland: 63°21'N	C. Bay leg. 1992
*Luzula spicata* (L.) DC	East Greenland: Godfred Hansen Ø	New north limit: 76°23'N	Bay 1992
*Luzula wahlenbergii* Rupr.	East Greenland: Dove Bugt	New north limit: 76°17'N	R. Corner and D. Shaw leg. 2006
*Melandrium affine* J Vahl coll.	East Greenland: Mt. Forel	New south limit: 66°46'N	Halliday 2019b
*Menyanthes trifoliata* L.	East Greenland: Jameson Land	New to East Greenland: 71°17'N	B. Fredskild and C. Bay leg. 1982
*Mertensia maritima* (L.) S. F. Gray	East Greenland: Zackenberg	New north limit in East Greenland: 74°28'N	C. Bay leg. 2005
*Minuartia biflora* (L.) Sch. and Th.	West and East Greenland	New north limits in West Greenland: 77°48'N and in East Greenland: 79°08'N	Bay 1992
*Minuartia groenlandica* (Retz.) Ostf.	West Greenland: Kitdlerngata	New north limit: 68°32'N	Fredskild 1996
*Minuartia stricta* (Sw.) Hiern	West and East Greenland	New north limits in West Greenland: 76°31'N and new south and north limit in East Greenland: 66°35'N and 77° 30'N, resp.	Bay 1992 Halliday 2019b
*Montia fontana* L.	East Greenland, Forsblads Fjord	New north limit in East Greenland: 72°25'N	R. and S. David leg. 1993
*Papaver cornwallisense* D. Löve	North and East Greenland	New to Greenland	H. Solstad pers. com.
*Papaver dahlianum* Nordh.	West, North and East Greenland	New to Greenland	H. Solstad pers. com.
*Papaver labradoricum* (Fedde) Solstad and Elven	West and East Greenland	New to Greenland	H. Solstad pers. com.
*Papaver lapponicum* (Tolm.) Nordh.	West Greenland	New to Greenland	H. Solstad pers. com.
*Pedicularis capitata* Adams	West Greenland	New south limit: 77°42'N	Bay 1992
*Pedicularis flammea* L.	West Greenland: Qaanaaq	New north limit: 77°28'N	F. Rune leg. 2017
*Pedicularis lapponica* L.	East Greenland: Bessel Fjord	New north limit: 75°58'N	G. Halliday leg. 1980
Pedicularis sudetica Willd. ssp. albolabiata Hult.	West Greenland: Thule district	New to Greenland: 77°28'N	Bay 1992
*Phleum commutatum* Gaud.	East Greenland: Jameson Land	New north limit: 70°58'N	Fredskild and Feilberg 1984
Phippsia algida ssp. algidiformis (H. Sm.) Löve and Löve	Northern Greenland	New subspecies to Greenland	Bay 1992
*Poa abbreviata* R.Br.	East Greenland: Kangerlussuaq	New south limit: 68°49'N	Halliday 2019b
*Poa alpina* L.	East Greenland: Nørlund Land	New north limit: 75°49'N	Halliday 2019b
*Poa flexuosa* Sm.	West Greenland	New to Greenland	Fredskild 1996
Poa hartzii Gandoger forma prolifera (Simm.) Boivin	North Greenland, Easternmost part	New forma to Greenland	Bay 1992
Poa pratensis L. ssp. colpodea (Th. Fr.) Tzvelev	East Greenland: Constable Pynt	New south limit: 70°44'N	Fredskild and Feilberg 1983
*Potamogeton filiformis* Pers.	East Greenland: Droning Louise Land	New north limit: 76°52'N	Bay 1992
*Potamogeton praelongus* Wulf.	West Greenland: Kangerdlugssuaq	New to West Greenland: 66°N	Bennike and Anderson 1998
Potamogeton pusillus L. ssp. groenlandicus (Hagstr.) Böch.	East Greenland: Skjoldungen	New to East Greenland: 63°21'N	C. Bay leg. 1992
*Potentilla stipularis* L.	East Greenland: Kuhn Ø	New north limit: 74°47'N	Bay 1992
*Primula stricta* Hornem.	East Greenland: Kuhn Ø	New north limit: 74°44'N	G. Halliday leg. 1990
*Puccinellia bruggemanni* Th. Sør.	North Greenland	New to Greenland	Bay 1992
*Puccinellia vaginata* (Lange) Fernald and Weath.	East Greenland: Bessel Fjord	New north limit: 75°58'N	G. Halliday leg. 1980
*Pyrola grandiflora* Rad.	East Greenland: Lindeman Fjord	New north limit: 74°40'N	K. Cartwright leg. 2002
*Pyrola minor* L.	West Greenland East Greenland: Milne Land	New north limit in West Greenland: 70°44'N New north limit in East Greenland: 70°44'N	Fredskild 1996 Corner leg. 1986
*Ranunculus auricomus* L. coll.	East Greenland: Clavering Ø	New north limit: 74°19'N	Halliday 2019a
*Ranunculus glacialis* L.	East Greenland: Nørre Mellemland	New north limit: 78°33'N	Bay 1992
*Ranunculus nivalis* L.	North Greenland: Peary Land	New north limit and new to North Greenland: 82°30'N	Bay 1992
*Ranunculus pygmaeus* Wbg.	East Greenland: Lambert Land	New north limit: 78°08'N	Bay 1992
*Ranunculus sabinei* R. Br.	East Greenland	New south limit: 74°51'N	Andersson leg. 1988
*Ranunculus subrigidus* W.B. Drew.	West and North Greenland	New to Greenland	R. Elven pers. com.
*Rumex acetosella*	East Greenland: Bessel Fjord	New north limit: 75°59'N	Halliday 2019a
*Sagina caespitosa* (J. Vahl) Lge.	West Greenland: Thule district; East Greenland: Kangerlussuaq	New north limit in West Greenland: 77°28'N New south limit in East Greenland: 68°09'N	Bay 1992 Kristine Westergaard leg. 2019
*Sagina procumbens* L.	East Greenland: Nørrefjord	New north limit in East Greenland: 71°08'N	B. Fredskild leg. 1985
*Sagina saginoides* (L.) Karst.	East Greenland: Jameson Land	New north limit: 70°47'N	R. Corner leg. 1986
*Salix glauca* L. coll.	West Greenland: Tugtuligssuaq	New north limit: 75°25'N	Bay 1992
*Saxifraga aizoides* L.	North Greenland: Campanula Dal	New north limit: 81°13'N	Bay 1992
*Saxifraga foliolosa* R. Br.	West Greenland: Narsaq East Greenland: Tasiilaq	New south limit in West Greenland: 61°N East Greenland: 65°35'N	Simonsen 2014; L. de Bonneval
*Saxifraga hieracifolia* W. and K.	West Greenland: Inglefield Land	New to West Greenland: 78°35'N	J. Feilberg leg. 1999
*Saxifraga hirculus* L.	East Greenland: Søndre Mellemland	New north limit: 78°05'N	Bay 1992
*Sibbaldia procumbens* L.	East Greenland: Kuhn Ø	New north limit: 74°44'N	Bay 1992
*Silene vulgaris* (Moench) Garcke	East Greenland: Tasiilaq	New to Greenland	Gartmann 1990
*Taraxacum brachyceras* Dahlst.	East Greenland: Kuhn Ø	New north limit: 74°44'N	G. Halliday leg.1990
*Taraxacum pumilum* Dahlst.	East Greenland: Shannon	New south limit: 75°11'N	Bay 1992
*Tofieldia coccinea* Richards.	North Greenland: Mylius Erichsen Land	New to North Greenland: 80°30'N	F. Daniëls leg. 1995
*Tofieldia pusilla* (Mixch) Pers.	East Greenland: Bessel Fjord	New north limit: 75°58'N	G. Halliday leg. 1980
*Trientalis europaea* L.	South Greenland	New to Greenland: 60°58'N	Bay 1993
*Triglochin palustris* L.	East Greenland: Tyroler Fjord	New north limit: 74°29'N	C. Bay leg. 2015
*Tripleurospermum maritima* (L.) W.D.J. Koch	East Greenland	New north and south limits: Revet (74°22'N); Sydkap (71°18'N).	Fredskild leg. 1991; Halliday 2019a
*Utricularia minor* L.	East Greenland: Jameson Land	New north limit: 71°33'N; New south limit: 71°06'N	R. Corner leg.; Bay and Fredskild 1982
*Vaccinium myrtillus* L.	East Greenland: Skjoldungen	New to East Greenland: 63°12'N	C. Bay leg. 1992
*Veronica officinalis* L.	East Greenland: Tasiilaq	New to Greenland	Gartmann 1990
*Woodsia alpina* (Bolt.) S. F. Grey	West Greenland: Melville Bugt East Greenland: Lyell Land	New north limit in West Greenland: 75°25'N; New north limit in East Greenland limit: 72°40'N	Bay 1992 R. and S. David leg. 1993
*Woodsia ilvensis* (L.) R. Br.	East Greenland: Hold With Hope	New north limit: 73°33'N	Hartz leg. 1891

## Annotated list of new vascular plant records for Greenland New species to the flora of Greenland since 1978

*Carex membranacea* Hook., *Carex miliaris* Michx, *Carex rhomalea* (Fernald) Mack.

During the revision of the *Carex saxatilis* complex a new species to Greenland, *Carex membranacea* Hooker, was found in Inglefield Land, Northwest Greenland, and in Fortune Bay in central West Greenland. Furthermore the complex was divided into three taxa by R. Elven: *Carex saxatilis* s.str., *Carex rhomalea* (Fernald) Mack., and *Carex miliaris* Michx. of which *Carex rhomalea* (Fernald) Mack., and *Carex miliaris* Michx. are new to the flora of Greenland.

*Equisetum hyemale* L.

The boreal species *Equisetum hyemale* was found in Tasiilaq district, Southeast Greenland (65°53'N) by Daniels and Van Herk (1984). The species is indigenous in Greenland and has probably immigrated to the east coast from Iceland by means of airborn spores.

*Festuca edlundiae* S. Aiken, Consaul and Lefkovich

Only one specimen of this recently described species is available in C. The collection is from Hold with Hope (73°28'N) in Northeast Greenland. Aiken et al. (2007) indicate that the species is found both in West and East Greenland. These specimens has not been included in the present study.

*Festuca groenlandica* (Schol.) Frederiksen

In Böcher et al. (1978)Festuca groenlandica is included as the var. groenlandica of *Festuca brachyphylla*. This taxon has been accepted at species level by Frederiksen (1982). *Festuca saximontana* Rydb.

Frederiksen (1982) published the find of this species as a new to Greenland and Fredskild (1996) mapped the distribution in Greenland between 64° and 70°N on the west coast.

*Galium verum* L.

This boreal species has been found once in Tasiilaq in Southeast Greenland (Gartmann 1990).

*Geum rossii* R. Br.

This species was found in Lambert Land (79°10'N) during the botanical mapping project in Northeast Greenland (Bay 1992). Only one individual of this Arctic-Alpine species was found in an open fell field vegetation

*Papaver radicatum* complex

Hitherto, the Greenlandic material of *Papaver* has been referred to *P. radicatum* Rotth. coll. but in the flora (Böcher et al. 1978) a few subspecies are mentioned. However, a taxonomical revision by Solstad and Elven concluded that the *Papaver radicatum* complex consist of four occurring in Greenland: *Papaver cornwallisense* D. Löve, *P. dahlianum* Nordh., *P. labradoricum* (Fedde) Solstad and Elven and *P. lapponicum* (Tolm.) Nordh. Consequently, *P. radicatum* Rotth. is excluded from the flora.

All four species occur in East Greenland although *P. cornwallisense* was only found once in the Scoresbysund area, whereas the other species are widespread (Solstad pers. com.). Pedicularis sudetica Willd. ssp. albolabiata Hult.

The first record of this species new to Greenland was collected in the vicinity of Qaanaaq (77°28'N) in northwest Greenland in 1975, but it was first correctly identified in 1985 as Pedicularia sudetica ssp. albolabiata by Halliday and Lang (1986). The species is only known from five localities in Thule district, Northwest Greenland (Bay 1992).

*Poa flexuosa* Sm.

This species was recognized and accepted as occurring in Greenland by Gjærevoll and Ryvarden (1977). Its present distribution in Greenland is from Ingitait Fjord (61°09'N) in Southeast Greenland to Ikorfat (70°45'N) in West Greenland. Nearly all collections in C and AAU are found at an altitude of 450–1400 m a.s.l. (Fredskild 1996).

*Puccinellia bruggemanni* Th. Sør.

In connection with the phytogeographical study of North Greenland (Bay 1992), the revision of the *Puccinellia angustata* material revealed 15 collections of a species new to Greenland: *Puccinellia bruggemanni* Th. Sør. The species was considered as endemic to the Canadian Arctic Archipelago (Aiken et al. 2007), now also including high arctic Greenland (Bay 1992).

*Ranunculus subrigidus* W.B. Drew.

R. Elven found this *Ranunculus* species new to the flora of Greenland among specimens from Northwest and North Greenland during a revision of *Ranunculus confervoides* Fr. specimens in C. *Silene vulgaris* (Moench) Garcke

This boreal amphi-atlantic species has been found once in Tasiilaq (66°N) in Southeast Greenland (Gartmann 1990).

*Trientalis europaea* L.

New to Greenland, found for the first time in 1992 in South Greenland (60°58'N) (Gartmann 1990). This boreal-alpine species is distributed mainly in Europe with its northern outpost in subarctic South Greenland and Iceland.

*Veronica officinalis* L.

This amphi-atlantic species has been found once in Tasiilaq in Southeast Greenland (66°N) (Gartmann 1990).

### New subspecies to the flora of Greenland

The subspecies Phippsia algida ssp. algidiformis (H. Sm.) Löve and Löve was found during the phytogeographical work in North Greenland (Bay 1992). It is distributed in high arctic Greenland from Scoresbysund (70°N) in East Greenland through North Greenland to the Thule district in Northwest Greenland (Bay 1992).

### New forma to the flora of Greenland

The viviparous form of Poa hartzii Gandoger forma prolifera (Simm.) Boivin has been found three times in Greenland. The localities are close to each other in the easternmost part of North Greenland. Two collections are from Kap København (82°23–24'N) in Peary Land and one from Prinsesse Margrethe Ø southeast of Peary Land. The form is described from material collected at Ellesmere Island and the only other collection outside

Greenland is from a small island near Devon Island (Scoggan 1978–1979). The distribution of *Poa hartzii* s.l. is Amphi-Atlantic high arctic.

### Species new to West Greenland

*Eriophorum callitrix* Cham.

First record is from Qaanaaq (77°28'N) in West Greenland collected by C. Bay in 1988.

*Potamogeton praelongus* Wulf.

First record in West Greenland at Kangerdlugssuak (66°32'N) by Bennike and Anderson (1998). The species is otherwise only known from Rypefjord (71°02) in central East Greenland.

*Saxifraga hieracifolia* W. and K.

This species was found in Inglefield Land (78°35'N) by F. Feilberg in 1999.

### Species new to East Greenland

*Carex chordorrhiza* Ehrh.

Until 1993 the species was only known from a few localities in southernmost Greenland (Feilberg 1984). A. Elvebakk found the species in Jameson Land (71°10'N) on the east coast in 1993 and C. Bay found the species further to the north at Tyroler Fjord (74°28'N) in 2015 – an extension of the distribution area of c. 350 kilometers. Fruits from the species are presumably transported to East Greenland by migrating geese from Iceland.

*Carex rostrata* Stokes

This species was found at one locality in Skjoldungen district (63°21'N) by C. Bay. It is the first record of *Carex rostrata* in East Greenland (Fredskild and Bay 1993).

*Diphasiastrum complanatum* (L.) Holub.

During fieldwork in 2014 C. Bay found one specimen of the species at Mudderbugten, Milne Land (70°35'N) in central East Greenland.

*Dryopteris assimilis* Walker

This species was collected at Fridtjof Nansens Halvø (64°20'N) by K. Gormsen in 1968. *Dryopteris filix-mas* (L.) Schott

This boreal species was found at three sites in Skjoldungen district (63°13'–21'N) during Greenland Botanical Surveys fieldwork in Southeast Greenland in 1992.

*Dupontia fisheri* R. Br.

The species was collected by B. Fredskild at Zackenberg (74°28'N) for the first time in East Greenland in 1992.

Isoëtes echinospora Dur. ssp. muricata (Dur.) Löve and Löve

The species was found by C. Bay at one locality (63°21'N) in Skjoldungen district.

*Juncus filiformis* L.

The species was found at three localities in Skjoldungen district between 63°16' and 63°21'N by Bay in 1992.

*Luzula parviflora* (Ehrh.) Desv.

The species was found at two localities in Skjoldungen district by C. Bay. The northernmost at 63°16'N

*Menyanthes trifoliata* L.

This species has been found for the first time on the west coast of Jameson Land (71°17'N) during the biological fieldwork prior to an oil exploration (Fredskild, Bay and Holt 1982). Totally, it was found in three lakes.

Potamogeton pusillus ssp. groenlandicus (Hagstr.) Böch.

This species was recorded once in Skjoldungen district (63°21'N) by C. Bay in 1992.

*Vaccinium myrtillus* L.

This species was found at Kap Niels Juel (63°12'N) by C. Bay and it is only the second record in Greenland. Hitherto it had only been found on the island Alangorssuaq in South Greenland.

### Species new to North Greenland

*Cardamine pratensis* L.

During fieldwork in eastern Peary Land (82°30'N) in 1987 and in Nansen Land (82°58'N) in 1991 collected the first records of the species in North Greenland (Bay 1992).

*Carex atrofusca* Schkuhr.

Finds by C. Bay in 1985 and 1991 in central North Greenland are the first records from North Greenland (Bay 1992). The species has a disjunct distribution in Greenland: In West Greenland it is recorded between Disko (69°16’ N and 71°30'N), in Northwest Greenland between Dundas (76°34'N) and Siorapaluk (77°48'N), plus the isolated records from Warming Land and Brainard Sund in North Greenland. In East Greenland it is distributed between Jameson Land (70°38’ N) and Lambert Land (79°10’ N).

*Carex glacialis* Mack.

The species was collected at 82°10'N in Wulff Land in 1985 by C. Bay.

Carex marina Dew. ssp. pseudolagopina (Th. Sør.) Böch.

Found at Brainard Sund (82°58'N) in Nansen Land which is the first record from North Greenland (Bay 1992). The only other find from North Greenland is from Mylius Erichsen Land (80°20'N) collected by Daniëls in 1995.

Dryas octopetala L. ssp. punctata (Juz.) Hult.

This species was collected in Amdrup Land (80°49'N) during the NEWland project in 1993 by C. Bay and B. Fredskild.

*Eriophorum callitrix* Cham.

C. Bay collected the species in Qaanaaq (77°28'N) in 1988 and in Nansen Land (82°58'N) in central North Greenland in 1991, which are the first records from West Greenland and a new north distribution limit in North Greenland.

Empetrum nigrum L. ssp. hermaphroditum

*Empetrum* was not known from North Greenland until F. Daniëls visited Mylius Erichsen Land (80°20'N) in 1995 and found a small population at Amdrup Højland. This is the first find in North Greenland, the previous northernmost record was Danmarkshavn (76°46'N) c. 500 km to the south.

*Ranunculus nivalis* L.

Collected by C. Bay at Kap København in eastern Peary Land (82°30'N), which is the first record from the flora province North Greenland (Bay 1992).

*Tofieldia coccinea* Richards.

F. Daniëls extended the known northern distribution limit in 1995 by finding the species for the first time in North Greenland at Mylius Erichsen Land at 80°30'N

North range extensions in West Greenland

*Antennaria canescens* (Lge. Malte)

Found in 1979 at Tugtuligssuaq, Melville Bugt (75°20'N) in northwest Greenland (Fredskild et al. 1979), which is an extension of 300 kilometers from Prøven (72°22'N) and in East Greenland the north range was extended to Kuhn Ø (75°38'N) by G. Halliday.

*Carex glacialis* Mack.

The flora states that it is recorded northward to 73°25'N in West Greenland and has an isolated record at Dundas (76°34'N). It is in addition recorded at six localities in North Greenland, the northern record at 82°10'N in Wulff Land.

*Carex lachenalii* Schkuhr.

According to Böcher et al. (1978) Upernavik (72°48'N) is the northernmost record in West Greenland. However the species is collected at four localities north of Upernavik: Tugtuligssuaq (75°25'N), Dunads (76°34'N), Qeqertat (77°30'N) and Etah, Inglefield Land; the new northern distribution limit at (78°18'N).

*Carex microglochin* Wbg.

The species was found at Svartenhuk (72°15'N) (Fredskild 1996).

Carex supina Wbg. ssp. spaniocarpa

Böcher et al. (1978) mentions Upernavik (72°47'N) as the northern limit, but it has been recorded four times northwards to Siorapaluk (77°48'N) (Bay 1992).

*Comarum palustris* L.

New north limit in Sydostbugten in West Greenland (68°40'N) (Fredskild 1996).

*Deschampsia alpina* (L.) R. and S.

New north limit in Sydostbugten in West Greenland at 68°37'N (Fredskild 1996).

Diapensia lapponica L. ssp. lapponica

North range extension from Upernavik (72°47'N) to Tugtuligssuaq (75°25'N) in 1979 (Fredskild et al. 1979) and further c. 200 kilometers to Qaanaaq (77°28'N) in 1988 (Bay 1992).

Eriophorum angustifolium Honk. ssp. subarcticum (V. Vassil.) Hult.

During GBS´ work in Northwest Greenland in 1979–1988 the species was found in several localities and the northern distribution limit was extended from 74°22'N to Qaanaaq (77°28'N).

*Eriophorum callitrix* Cham.

New to West and North Greenland (Bay 1992). It has isolated occurrences in Thule district and Nansen Land collected by C. Bay in 1988 and 1991, respectively.

*Juncus trifidus* L.

New north limits in West Greenland at 72°40'N (Fredskild 1996).

Ledum palustre L. ssp. decumbens (Ait.) Hult.

The species was known from West Greenland between 62°54'N and 72°51'N before it was found at McCormic Ford in Thule district (77°41'N), which extended the northern distribution limit by c. 500 km.

*Pedicularis flammea* L.

Bay found it during fieldwork at Moriusaq (76°45'N), West Greenland in 1988 (Bay 1992) and Rune (pers. com.) found it further to the north at Qaanaaq 77°28'N in 2017.

*Pyrola minor* L.

New northern distribution limit at Disko island (70°44'N) in West Greenland.

*Puccinellia vaginata* (Lange) Fernald and Weath.

New north limit at 75°58'N in West Greenland (Fredskild et al. 1982).

*Sagina caespitosa* (J. Vahl) Lge.

North range extension in West Greenland from Upernavik (72°47'N) to Tugtuligssuaq (75°25'N) in 1979 and further to Qaanaaq (77°28'N) in 1988 (Bay 1992).

*Salix glauca* L. coll.

Found in 1979 at Tugtuligssuaq, Melville Bugt (75°25'N) (Fredskild et al. 1979). Previous northernmost record was at 74°30'N.

*Woodsia alpina* (Bolt.) S. F. Grey.

This species was found at Tugtuligssuaq (75°25'N) in 1979, which is a new north limit for West Greenland (Bay 1992).

### North range extensions in East Greenland

*Arabis arenicola* (Richards) Gel.

Found further to the north at 74°38'N at Lindeman Fjord by G. Halliday in 1980, an extension of c. 200 kilometers to the north (Halliday 2019b).

*Botrychium lunaria* (L.) Swe.

O. Gilg collected this species in 2005 on Gaus Halvø (73°18'N), which is a new north limit in East Greenland (Gilg 2005). Halliday (2019a) mentions Shaws collection in 2007 from Ella Ø as the northernmost locality in East Greenland (72°47'N).

*Carex macloviana* D´Urv.

Recorded at Adolf S. Jensen Land (75°25'N) in East Greenland by Corner in 2008 as new north limit. The species was hitherto only known from one locality north of Mestersvig (72°14'N) in East Greenland at Myggbukta (73°31'N) collected by Corner in 2001.

*Carex norvegica* Retz.

New north limit in East Greenland at Femdalen, Ostenfeldts Land (75°18'N).

*Carex parallela* (Læst.) Sommerf.

Corner collected the species at Adolf S. Jensen Land (76°16'N) in 2006, c 100 kilometers north of the known northern distribution limit.

*Carex scirpoidea* Michx.

The species was found at Kuhn Ø (74°46'N) in East Greenland by R. Corner and G. Halliday in 1990.

*Cerastium cerastoides* (L.) Britton

New north limit in East Greenland at Lindemann Fjord (74°38'N) collected by G. Halliday (2019b).

*Deschampsia pumila* Ostenf.

Found at Norske Øer (79°03'N) during the biological mapping project in 1989–1990, which is a new north limit in East Greenland (Bay 1992).

*Draba fladnizensis* Wulf.

In 1990 it was found at Lambert Land extending the northern distribution limit to 79°08'N (Bay 1992).

*Dryopteris assimilis* Walker

New north limit in Skjoldungen district (64°20'N).

*Elymus hyperarcticus* (PoluN) Tzvel.

During the environmental survey prior to an oil exploration in Jameson 1982–1985 the species was found as far south as (70°44'N), which is a new south distribution limit.

*Galium triflorum* Michx.

New north limit in East Greenland at 63°28'N (Fredskild et al. 1992).

*Gentiana detonsa* Rottb.

R. and S. David collected the species at Tyroler Fjord (74°30'N). This is the northernmost collection in Greenland.

*Gymnocarpium dryopteris* (L.) Newman

Fredskild found *Gymnocarpium dryopteris* at a hot spring in Liverpool Land (71°08'N) in central East Greenland (Fredskild et al. 1987). This is an extension of the northern distribution limit by more than 200 km.

*Juncus ranarius* Perr. and Song.

The collection from 1985 by Fredskild from Liverpool Land (71°08'N) in central east Greenland is a new northern record. Hitherto the species is not found north 69°22 ´N *Juncus trifidus* L.

New north limit in East Greenland at Kuhn Ø (74°44'N) collected by G. Halliday (2019b).

*Luzula wahlenbergii* Rupr.

The species was only known from Zackenberg (74°28'N) until the intensive botanical investigations took place in East Greenland in 1989–1990 (Fredskild and Bay 1990, 1991). The species was found at another ten localities (74°28'–75°55'N) extending the northern distribution limit to Bessel Fj. (75°55'N) (Bay 1992). Corner and Shaw found it further to the north at 76°17'N in 2006.

*Mertensia maritima* (L.) S. F. Gray

The species is mainly distributed in the Disko-Nugssuaq (68°–72°30'N) in West Greenland and known from Thule district on the west coast. It was found at Zackenberg research station (74°28'N) in East Greenland in 2005 and disappeared few years later. It was presumably brought from West Greenland by a researcher who had worked in central West Greenland before appearing in East Greenland. In East Greenland it is only known from Tasiilaq (66°N) and Skjoldungen (63°N) in addition to Zackenberg.

*Minuartia biflora* (L.) Sch. and Th.

Found at all localities explored in 1979 and 1988 in Northwest Greenland (Fredskild and Bay 1990) and East Greenland in 1989–1990 (Fredskild and Bay 1991, Bay 1992). The known distribution limit is now at 77°48'N in Northwest Greenland and 79°08'N in Northeast Greenland (Bay 1992).

*Minuartia stricta* (Sw.) Hiern

The distribution is extended both in West and East Greenland. The species is distributed between 66°20'N and Dundas (76°31'N) in West Greenland (Fredskild 1996) and from Tasiilaq district (66°35') (Halliday 2019a) to Skærfjorden (77°35'N) in East Greenland (Bay 1992).

Montia fontana L. ssp. fontana

R. and S. David collected *Montia fontana* at Forsblads Fjord (72°25'N) in 1993, 700 kilometers north of the hitherto northernmost record in East Greenland at 66°04'N *Pedicularis flammea* L.

C. Bay collected the species at Nordre Mellemland (80°38'N) in 1990, which is a new northern distribution limit (Bay 1992).

*Phleum commutatum* Gaud.

New north limit at Jameson Land (70°58'N) recorded by C. Bay in 1983 (Fredskild 1984).

*Pyrola minor* L.

New northern distribution limit at Milne Land (70°04'N) by C. Bay in 2014.

*Poa alpina* L.

New north limit at 75°49'N (Halliday 2019b)

*Potamogeton filiformis* Pers.

Until 1989, when it was found at Droning Louise Land (76°52'N) during the biological mapping of East Greenland, the species was recorded northwards in East Greenland to Clavering Ø (74°20'N).

*Potentilla stipularis* L.

New north limit at Kuhn Ø (74°47'N) (Bay 1992).

*Primula stricta* Horn

Found on Kuhn Ø during the British North-east Expedition 1990 (Halliday and Corner 1991); an extension of the north limit of c. 100 kilometers.

*Pyrola grandiflora* Rad.

New north limit at 74°40'N collected by Cartwright in 2002.

*Pyrola minor* L.

New northern distribution limit at 70°44'N in Sødal, where the species was found by Corner in 1986.

*Ranunculus auricomus* L. coll.

New north limit at 74°19'N in East Greenland (Halliday 2019a).

*Ranunculus glacialis* L.

The species was found at Nørre Mellemland (78°33'N) in Northeast Greenland in 1989, which is a new northern distribution limit (Bay 1992).

*Ranunculus pygmaeus* Wbg.

New north limit at Lambert Land (78°08'N) collected by C. Bay and Fredskild in 1990 (Bay 1992).

*Sagina procumbens* L.

Found at a hot spring at Nørrefjord, Liverpool Land (71°08'N) during fieldwork of Greenland Botanical Survey in 1985; hitherto not found north of Knighton Bugt (69°22'N) on the east coast.

*Sagina saginoides* (L.) Karst.

Found in Jameson Land (70°47'N) by Corner in 1986. Previous northernmost record is at Rømer Fjord (69°43'N).

*Saxifraga hirculus* L.

It was found during the biological mapping of Northeast Greenland in 1990 at Søndre Mellemland (78°05'N) (Bay 1992).

*Sibbaldia procumbens* L.

The northern limit was extended by c. 200 kilometers to Kuhn Ø (74°44'N) during the British North-east Expedition in 1990 (Halliday and Corner 1991).

*Triglochin palustre* L.

C. Bay found this species at Tyroler Fjord (74°30'N) in 2015, which is the northernmost record in East Greenland. *Woodsia alpina* (Bolt.) S. F. Grey.

Halliday (2019a) mentions it from Lyell land (72°40'N), which is a new record for East Greenland.

*Woodsia ilvensis* (L.) R. Br.

Böcher et al. (1978) states that the species is not recorded north of 71°N in East Greenland. However, a specimen from Hold with Hope (73°33'N) by Hartz from 1891 is the northernmost collection from East Greenland.

### North range extensions in North Greenland

*Arenaria pseudofrigida* (Ostf. and Dahl) Juz.

Found at Brainard Sund, Nansen Land (82°58'N) in North Greenland, which is a new north limit (Bay 1992).

*Arnica angustifolia* M. Vahl

This species has been found four times in central north Greenland; northernmost find at 83°04'N collected by C. Bay in 1985 (Bay 1992).

*Carex glacialis* Mack.

The species was collected at 82°10'N in Wulff Land in 1985 by C. Bay.

*Carex rupestris* All.

New north limit at Brainard Sund (82°58'N) in North Greenland collected by Bay (1992).

*Epilobium arcticum* Sam.

F. Schwartzenbach collected the species at Frigg Fj. (83°15'N), this is only the third find in the flora province North Greenland. Previously, it has been collected at Centrum Sø (80°10'N).

*Juncus castaneus* Sm.

New north limit at Brainard Sund, Nansen Land (82°58'N), which is only the sixth record from North Greenland (Bay 1992).

*Kobresia simpliciuscula* (Wbg.) Mack.

New to western North Greenland. Hitherto only known from four localities in North Greenland (Bay 1992). It was collected at two localities in Warming Land in central North Greenland in 1985 (Aastrup et al. 1986).

*Saxifraga aizoides* L.

New northern distribution limit at 81°13'N in Mylius Erichsen Land.

### South range extensions in West Greenland

*Antennaria angustata* Greene

The species was found at Isua (65°12'N) in West Greenland by Bay and Simonsen (2013).

*Braya thorild-wulffii* Ostf.

The species was found on the east coast of Disko Island 69°54'N (Fredskild 1996).

*Carex holostoma* Drej.

Found by C. Bay during fieldwork in 2009 and 2013 south of the southern distribution limit. The southernmost find is at 65°32'N.

*Carex misandra* R. Br.

The new south record at Isua in West Greenland (65°12'N) was recorded in 2012 (Bay and Simonsen 2013).

*Carex rupestris* All.

This northern species has extended the southern distribution limit to 63°59'N in West Greenland.

*Draba cana* Rydberg

Found at Narsaq (61°N) by Simonsen in 2014. This is an extension of the south distribution limit by 400 kilometers.

*X Ledodendron vanhoeffeni* (Abromeit) Dalgaard and Fredskild.

The species was only known from one locality in central West Greenland (Böcher et al. 1978), but the knowledge of the distribution has been extended by eight finds in central West Greenland southward to 66°30'N (Dalgaard and Fredskild 1993).

*Luzula arctica* Blytt

This northern distributed species was found at a new southern distribution limit in West Greenland during fieldwork in the mining area Isua in West Greenland (Bay and Simonsen 2013).

### South range extensions in East Greenland

*Calamagrostis purpurascens* R. Br.

The species was found at six localities during a Greenland Botanical Surveys expedition to Skjoldungen district in 1992. The southernmost record was at 63°21'N and it is an extension of the distribution area of c. 700 km to the south.

*Draba fladnizensis* Wulf.

The find of *Draba fladnizensis* in Skjoldungen district (63°28'N) was an extension of the species east Greenland distribution to the south; hitherto known from East Greenland between Jameson Land (70°N) and Lambert Land (79°10'N).

*Elymus hyperarcticus* (PoluN) Tzvel.

The species was collected in Jameson Land (70°44'N) by B. Fredskild and C. Bay in 1982. The hitherto southernmost record was at Botanikerbugt, Ymer Ø (73°10'N).

*Galium brandegei* A. Gray.

Found in Southeast Greenland (63°21'N) by C. Bay in 1992. This is the first find on the east coast between South Greenland and Tasiilaq.

*Melandrium affine* J Vahl

New south limit at Mt. Forel (66°39'N) in East Greenland (Halliday 2019b).

*Poa abbreviata* R.Br.

Found at Kangerlussuaq in East Greenland (68°49'N), which is a new south limit (Halliday 2019b).

Poa pratensis L. ssp. colpodea (Th. Fr.) Tzvelev

New south limit at Constable Pynt (70°44'N).

*Ranunculus sabinei* R. Br.

New south limit at 74°51'N found by Hauge Andersson in 1988.

*Sagina caespitosa* (J. Vahl) Lge.

K. Westergaard found the species at Kangerlugssuaq (68°09'N) in East Greenland in 2019.

*Saxifraga foliolosa* R. Br.

New south limit at Tasiilaq (65°35'N) in Southeast Greenland collected by M. Strandberg.

*Taraxacum pumilum* Dahlst.

New south limit at Shannon at 75°11'N (Bay 1992).

*Corrections to the Flora of Greenland* (Böcher et al. 1978)

During the study of the vascular plant flora of East Greenland a few corrections to the flora of Greenland (Böcher et al. 1978) showed up.

*Callitriche anceps* Fern

No collections from Skjoldungen district have been found in herb. C, BM, LANC or O as stated in the flora of Greenland. Consequently, the species is strictly distributed at the west coast of Greenland.

*Callitriche palustris* L.

Northernmost record is from 71°17'N in East Greenland and not as stated in the Flora of Greenland at 72°40'N.

*Hippuris vulgaris* L.

Recent studies show that *Hippuris lanceolata* Retz. is the most common species in the Western Arctic (Elven et al. 2012). It is the only species documented from Greenland and the Canadian Arctic Archipelago; its two relatives (*Hippuris tetraphylla* L. f. and *H. vulgaris* L.) occur together with H. lanceolata on the American mainland, but not in Greenland.

*Honckenya peploides* (L.) Ehrh.

The northernmost locality is Siorapaluk (77°48'N) in West Greenland (Fredskild and Bay 1988) and Mørkefjord (77°55'N) in East Greenland. No specimens of *Honckenya peploides* has been found in Inglefield Land, which is the northern limit according to Böcher et al. (1978).

*Subularia aquatica* L.

No evidence has been found of *Subularia aquatica* L. occurring at 69°43'N in East Greenland. Consequently, the northernmost find in East Greenland is at 65°39'N

*Woodsia ilvensis* (L.) R. Br.

Böcher et al. (1978) states that the species is not recorded north of 71°N in East Greenland. However, a specimen from Hold with Hope (73°33'N) by Hartz from 1891 is the northernmost collection from East Greenland.

## Conclusions

### The number of vascular plant taxa in the flora of Greenland

The latest update of the number of vascular species of the flora of Greenland, which is 25 years old (Bay 1993) summarized the total number to 513. During the latest 25 years the total number of vascular plants has been increased by twenty species giving a total of 532 species. Holt et al. (2019) recently presented a photo flora on the internet totaling 625 species of vascular plants including introduced species and planted tree species.
